# Mediating Roles of hsCRP, TNF-α and Adiponectin on the Associations between Body Fat and Fatty Liver Disease among Overweight and Obese Adults

**DOI:** 10.3390/biology10090895

**Published:** 2021-09-10

**Authors:** Ming Xie, Haokai Tang, Feifei Li, Si Wu, Yanhui Dong, Yide Yang, Julien Steven Baker, Jun Ma

**Affiliations:** 1 Key Laboratory of Molecular Epidemiology of Hunan Province, School of Medicine, Hunan Normal University, Changsha 410081, China; zhongyan@hunnu.edu.cn (M.X.); tanghk@hunnu.edu.cn (H.T.); 201930194058@hunnu.edu.cn (S.W.); 2 Centre for Health and Exercise Science Research, Hong Kong Baptist University, Kowloon Tong, Hong Kong 999077, China; lifeifei@hkbu.edu.hk; 3 Department of Sport, Physical Education and Health, Hong Kong Baptist University, Kowloon Tong, Hong Kong 999077, China; 4 Institute of Child and Adolescent Health, School of Public Health, Peking University Health Science Center, Beijing 100191, China; majunt@bjmu.edu.cn

**Keywords:** inflammatory factor, obesity, mediation analysis, body fat percentage, gender difference

## Abstract

**Simple Summary:**

Body fat has been reported to be related to a higher risk of fatty liver disease (FLD). However, few studies have explored the mediating roles of inflammatory biomarkers or adipokines on the relationships. This study examined the potential mediating effects of high sensitivity C-reactive protein (hsCRP), tumor necrosis factor-α (TNF-α) and adiponectin (APN) in relationships between body fat and FLD in overweight and obese adults. Additionally, gender and age differences were demonstrated. It was concluded that hsCRP has a significant mediating effect on the association between body fat percentage and FLD in females independent of potential covariates. It was also demonstrated that the mediation effect of hsCRP was only significant and more profound in relatively older adults (36–56 years, 38.3%), not significant in the young ones (19–35 years). TNF-α and APN were not significantly associated with body fat percentage or FLD, with no mediating effect on the association between body fat percentage and FLD observed in either gender. In conclusion, hsCRP was a potential mediator on the association between adiposity and FLD, and this mediation is gender-specific and age-specific. The authors hope that the findings could contribute to the further exploration of the inflammatory-related mechanism of obesity-associated FLD.

**Abstract:**

Body fat has been reported to be associated with a higher risk of fatty liver disease (FLD). However, few studies have explored the mediating roles of an inflammatory biomarker or adipokine on the relationships. Here, we examined the potential mediating roles of high sensitivity C-reactive protein (hsCRP), tumor necrosis factor-α (TNF-α) and adiponectin (APN) in relationships between body fat and FLD in overweight and obese adults. Additionally, gender differences will be investigated. In total, 1221 participants aged 19–56 years were included in our study. Body fat percentage was measured with Dual Energy X-ray Absorptiometry (DEXA) and FLD by abdominal ultrasound. Mediation analysis was performed to assess the mediating effect of hsCRP, TNF-α and APN on the associations between BF (%) and FLD by gender differences. We found that hsCRP was significantly associated with body fat percentage in both genders (b = 0.2014, *p* < 0.0001 and b = 0.1804, *p* < 0.0001 for male and female, respectively), while hsCRP was associated with FLD only in the female group (b = 0.1609, *p* = 0.0109) but not in male group (b = 0.4800, *p* = 0.0603). We observed that hsCRP has a significant mediating effect on the association between body fat percentage and FLD (b = 0.0290, *p* = 0.0201, mediation ratio: 13.6%) in the female group independent of potential covariates (age, smoking, alcohol drinking and physical activity). TNF-α was not significantly associated with body fat percentage or FLD, with no mediating effect on the association between body fat percentage and FLD in either gender. In conclusion, there is a gender-specific mediation role of hsCRP in the association between body fat and FLD. HsCRP was a potential mediator on the association between adiposity and FLD in the female gender, but not in the male gender. Higher body fat was associated with a higher risk of FLD, and the inflammation level might play a potential mediating role in the association between body fat and FLD among female overweight and obese adults.

## 1. Introduction

Fatty liver disease (FLD), which is defined as lipid accumulation exceeding the normal range of 5% of liver wet weight, often occurs in patients with other conditions, such as obesity and type 2 diabetes [1]. FLD is usually classified as nonalcoholic fatty liver disease (NAFLD) and alcoholic liver disease (ALD) [2,3]. A previous study suggests that being overweight plays an even greater role in liver fat accumulation than heavy drinking [4]. From the global perspective, it was estimated in 2018 that about 25% of the world population had NAFLD [5]. The prevalence of FLD in China was estimated to be 16.73% (95%CI: 13.92–19.53%) in a systematic review in 2015 [6]. Notably, a recent meta-analysis reported that the prevalence of NAFLD in China was 29.2% in 2018 [7], much higher than even the same year global prevalence of NAFLD (25%) [8]. In recent years, with changes in lifestyle and dietary habits, the incidence of FLD has risen sharply. The prevalence of FLD ranged from 12.5% to 27.3% [9,10], becoming the most common chronic liver disease in China [11]. Therefore, it is important to identify risk factors or correlates for FLD in Chinese populations.

Accumulating evidence has shown that FLD is closely related to obesity and fat accumulation, especially for NAFLD [2,12,13,14]. Numerous epidemiology and animal studies have indicated that a higher body fat percentage is significantly associated with an elevated risk of FLD [15,16,17,18]. However, until now, the pathogenesis of obesity-related FLD remains unclear. Previous researchers speculated several hypotheses of mechanisms of obesity-related FLD progression, including insulin resistance, low-grade inflammation and intestinal microbiota, etc. [19,20,21,22]. Among these mechanisms, one important potential mechanism is chronic low-grade inflammation status induced by abnormal or excessive body fat accumulation [21,22]. In reality, adipose tissue is not only a main place to store excessive energy but also an important but usually neglected endocrine organ [21]. It can secrete a number of adipokines, including pro-inflammatory biomarkers (tumor necrosis factor-α, TNF-α, etc.) and anti-inflammatory biomarkers (Adiponectin, APN, etc.), and also it can indirectly regulate the inflammation status (C-reaction protein, CRP) [23]. Pro-inflammatory cytokines/chemokines are considered to be important biomarkers of adipose inflammation. Tumor necrosis factor-α (TNF-α) is an effective pro-inflammatory cytokine secreted by the macrophages and adipocytes, especially visceral adipose tissue. These cytokines are directly involved in insulin and glucose metabolism, and induce insulin resistance and stimulate lipolysis [24]. The liver is an important secretion organ for biomarkers of inflammation and endothelial dysfunction. TNF-α can induce the expression of high sensitivity C-reactive protein (hsCRP), fibrinogen and other acute-phase proteins [25,26]. It has been demonstrated that patients with NAFLD have elevated levels of fibrinogen and hsCRP [27]. In addition, previous studies have shown that TNF-α plays an important role in the development and progress of FLD [28,29]. Adiponectin is an anti-inflammatory adipocytokine. In the liver, adiponectin acts by activating 5-AMP-activated protein kinases and peroxisome proliferator-activated receptor-α pathways, as well as inhibiting Toll-like receptor-4 mediated signaling [30]. There is evidence that adiponectin decreases hepatic and systemic IR and attenuates liver inflammation and fibrosis [31,32].

The adipose tissue depots of overweight and obese participants are usually expanded, coupled with hyperplasia and hypertrophy [33]. When adipose tissue expands, there is a sustained inflammatory response, accompanied by adipokine dysregulation, leading to chronic subclinical inflammation and insulin resistance [34]. On the other hand, the relationships between TNF-α, APN, hs-CRP and FLD are also demonstrated in epidemiology studies and animal studies [13,35,36,37,38,39]. However, inflammation status as a potentially important link between obesity and FLD and the specific mediation role or effect of key inflammatory biomarkers (hs-CRP, TNF-α and APN) between the association of obesity and FLD have not been explored.

**Figure 1:**
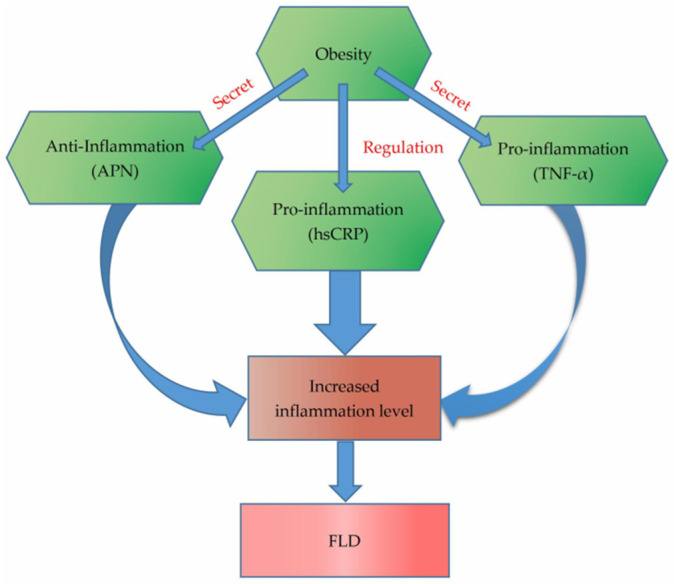
The relationship between obesity and fatty liver disease.

Therefore, we hypothesize that hsCRP, TNF-α and APN might function as mediation factors in the association between adiposity and risk of FLD (Figure 1), and considering the sex difference of fat distribution and risk of FLD [40,41], we speculate that the mediating effect might be gender-specific. To test the hypothesis, this present study aimed to demonstrate the mediation roles of APN, TNF-α and hsCRP on the relationships between body fat and FLD, as well as the possible gender differences.

## 2. Materials and Methods

### 2.1. Participants

**Figure 2:**
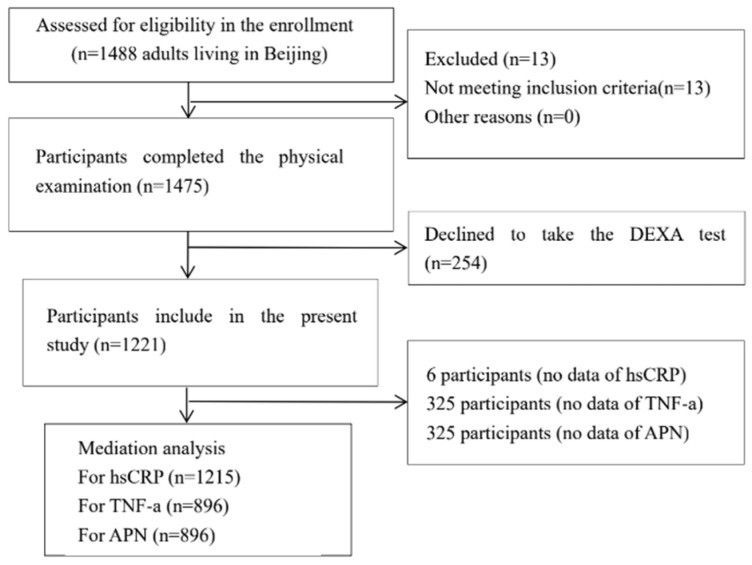
Flowchart of the study sample selection (dual-energy X-ray absorptiometry, DEXA; hsCRP, high sensitivity C-reactive protein; TNF-α, tumor necrosis factor-α; APN, adiponectin; FLD, Fatty liver disease).

Chinese residents living in Beijing for ≥1 year, aged 19–56 years old, and categorized as overweight or obese were invited to participate in this study. In relation to the inclusion and exclusion criteria, finally, one thousand and two hundred twenty-one participants were included (Figure 2) [41]. This study complied with the Institutional Review Board Statement: The study was conducted according to the guidelines of the Declaration of Helsinki and approved by the Peking University Medical Ethics Committee (IRB00001052-13086). All subjects signed and provided informed consent forms. Date of birth, physical activity level, alcohol consumption and cigarette smoking status were investigated using a questionnaire [41]. Moderate-intensity physical activity was categorized as no (exercise less than once per week) and yes (exercise more than once per week); high-intensity physical activity was categorized as no (exercise less than once per week) and yes (exercise more than once per week) [42].

### 2.2. Anthropometric Measurements

Height and weight were measured by trained and experienced investigators (all were senior medical students) with standardized protocols, which have been described previously [43]. We measured weight using a standard lever scale to the nearest 0.1 kg; height by a stadiometer to the nearest 0.1 cm. The participants were required to wear light clothing, be barefoot and stand upright, daily calibration of the instrument was performed prior to use. All measurements were conducted twice, and the mean value was used for the final analysis.

### 2.3. Laboratory Measurements

Blood samples from participants after 8 h of fasting were collected and separated into serum for further analysis. The serum hsCRP levels were measured using immune-turbidimetric assay (Automatic biochemical analyzer AU400, OLYMPUS, Tokyo, Japan) and TNF-α was quantified via enzyme-linked immunosorbent assay (ELISA) reagent kits (R&D Systems Inc., Minneapolis, MI, USA) following the manufacturer’s instructions. Intra-class correlation coefficients [ICC] for the tests are all >0.75, indicating the laboratory tests are relatively stable and reliable. The reference values of hsCRP, TNF-a and adiponectin were 0.02–156 mg/L, 1.6–1000 pg/mL and 10–20,000 ng/mL.

### 2.4. Body Fat Percentage Measurement

Body fat percentage was measured by dual-energy X-ray absorptiometry (DEXA) method (GE Healthcare, Lunar iDXAME +210205, Madison, WI, USA). The measurement was conducted by a professional and experienced clinical doctor in the hospital according to a standardized protocol.

### 2.5. Diagnostic Criteria of FLD

The diagnosis of fatty liver was based on the results of real-time ultrasonography (Logiq 180, GE, Wauwatosa, WI, USA) with standard criteria measured by experienced sonographers. The images of the liver were assessed to be normal if the liver exhibited fine level echoes, and was oechoic compared with renal cortex, adequate visualization of the hepatic vessels and diaphragm, and also the texture of liver was homogenous. Fatty liver was diagnosed according to the criteria of the presence of at least 2 of 3 abnormal findings in the abdominal area: (1) diffusely increased echogenicity liver (bright liver with its echogenicity greater than the kidney); (2) vascular blurring; (3) gradual attenuation of ultrasound echo [44]. 

Specifically, hepatorenal echo contrast was diagnosed with the evidence of sonographic contrast between the right kidney cortex along the midaxillary line and the liver. Bright liver was diagnosed by abnormally high-level and intense echoes from the liver parenchyma, and gradual or even deep attenuation was identified by evident attenuation of echo penetration into the deep liver part and impaired or abnormal diaphragm visualization25. Vascular blurring was diagnosed by an impaired or abnormal visualization of the intrahepatic vessels borders and their lumen getting narrow [45].

The sensitivity and specificity of abdominal ultrasonography in the diagnosis of FLD were 92% and 100%, respectively [46].

### 2.6. Statistical Analysis

**Figure 3:**
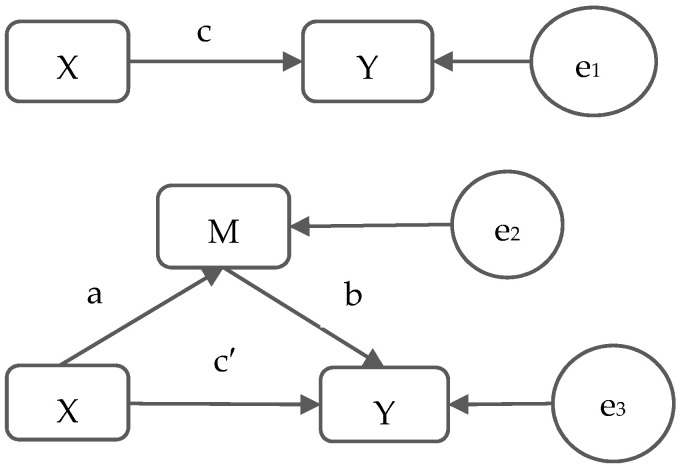
Model of mediation analysis. X means the independent variable (BF(%)), M means the potential mediator (hsCRP or TNF-α), and Y means the dependent variable (FLD). Association c: association between the independent variable (X) and dependent variable (Y). Association a: association between X and potential mediation variable (M). Association b: association between M and Y after controlling for X. Association c′: association between X and Y after controlling for M.

SPSS for Windows (version 20.0, SPSS Inc., Chicago, IL, USA) was used for statistical analysis. The basic characteristics of the participants were described with median (interquartile ranges, IQR), including age, height, weight, BMI, fat mass, skeletal muscle mass, BF (%), hsCRP, TNF-a, and APN. For the continuous variables Mann–Whitney U tests were performed for between-group comparisons. We conducted chi-square tests to compare the categorical variables. The mediating effects of serum hsCRP, TNF-α or APN on the relationship between body fat percentage and FLD were analyzed by SPSS mediating analysis procedures provided by Andrew F. Hayes (http://www.afhayes.com/, accessed on 23 May 2015) [47]. The proposed mediator (M), independent variable (X) and dependent variable (Y) were standardized to make comparisons. The process of mediation effect analysis was developed according to the mediation theory of Baron R.M. and Kenny D.A. [48]. Firstly, we used logistic regression analysis to test the total effect (c) of the independent variable X on the dependent variable Y, and then tested the effect (a) of X on the potential mediator M. Subsequently, the effect of M on Y after controlling X (b) and the effect of X and Y after controlling M (c′) were analyzed. If the three coefficients (a, b and c) were statistically significant, then we calculated the product of a and b (indirect effect, a × b) as the mediating effect of M, and performed the Sobel test to test the statistical significance of mediating effect (a × b). The ratio or proportion of mediation effect is the percentage of potential mediation effect in the total effect (a × b/c). The statistical model of mediation analysis is shown in Figure 3. Additionally, we conducted the mediation analysis with further adjustment of skeletal muscle mass as the sensitivity analysis.

## 3. Results

### 3.1. Basic Characteristics of Participants

One thousand and two hundred twenty-one subjects who were overweight or obese were involved in the current study. Table 1 outlines the basic characteristics of the subjects in this study. The median of age, body mass index (BMI) and BF (%) was 33 years vs. 37 years, 29.4 kg/m^2^ vs. 28.0 kg/m^2^, 33.8% vs. 41.5% for male and female participants, respectively. Significant gender differences in age, body fat percentage, weight, skeletal muscle mass and BF (%) than the males (*p* < 0.001). Men had higher weight, height, skeletal muscle mass and BMI than women (*p* < 0.001). The age, APN and body fat percentage levels of women were higher than men (*p* < 0.001). 

### 3.2. Relation between Body Fat and Proposed Mediators (a)

Associations between body fat percentage, proposed mediators (including hsCRP, TNF-α and APN), and FLD are shown in Table 2. For hsCRP, both in males and females, hsCRP were significantly associated with BF (%) (a = 0.2014, SE = 0.0439 and a = 0.1804, SE = 0.0295 for males and females, respectively, *p* < 0.0001). In both males and females, TNF-α and APN were not significantly associated with BF (%) (*p* > 0.05).

### 3.3. Relation between Proposed Mediators and FLD (b)

With adjustment of the potential covariates, hsCRP level was significantly related with FLD in the total population (b = 0.1812, SE = 0.0625, *p* = 0.0037). This association was not statistically significant in males (*p* > 0.05), while in females, hsCRP was significantly related with FLD (b = 0.1609, SE = 0.0632, *p* = 0.0109). In both males and females, TNF-α and APN was not significantly related with FLD (*p* > 0.05).

### 3.4. Mediation Effect of Proposed Mediators (Indirect Effect, a × b)

We performed mediating analysis to test the mediating effect (indirect effect, a × b) of hsCRP, TNF-α and APN. The results of the mediation analysis of hsCRP, TNF-α and APN are shown in Table 2. In the total population, we found hsCRP had a significant mediating effect on the association between body fat percentage and FLD (c′ = 0.0335, SE = 0.0125, *p* = 0.0072, mediation ratio: 14.8%). When stratified by gender, we found that the mediating effect was only significant in female participants (c′ = 0.0290, SE = 0.0125, *p* = 0.0201, mediation ratio: 13.6%) but not significant in males (*p* > 0.05). When stratified by age group, the significant mediating effect was observed only in participants 36–56 years (c′ = 0.0778, SE = 0.0288, *p* = 0.0069, mediation ratio: 38.3%, Table 3).

For TNF-α and APN, no significant mediating effect on the association between body fat percentage and FLD were found in males or females, young adults or older adults (Table 2 and Table 3). Additionally, in the sensitivity analysis with further adjustment of skeletal muscle mass, similar results were observed for the mediation effect of hsCRP on the relationship between BF (%) and risk of FLD (data not shown).

## 4. Discussion

Our study found significant gender differences regarding the association between hsCRP and risk of FLD in overweight or obese adults; with adjustment of body fat, only in female participants was hsCRP significantly associated with FLD. Subsequently, we observed a significant mediation effect of hsCRP on the association between body fat and FLD which existed only in female subjects, and no significant mediation effects of TNF-α or APN were observed in both genders. However, hsCRP explains only a small proportion of the relationship between body fat and FLD (Total population: 14.8%, Female: 13.6%, 36–56 years: 38.3%). These findings imply hsCRP as a potential pro-inflammatory factor contributing to the development of obesity-related FLD in females and the underlying mechanisms for adiposity-related FLD are likely to be different between females and males.

Our results provided strong positive associations between the pro-inflammatory biomarker (hsCRP) and body fat in both females and males, which is consistent with previous studies [49,50]. In our study, we used DEXA to measure body fat, which is more precise and accurate than BMI [51] or waist-to-height ratio [52] as used in previous studies. Therefore, this study would potentially provide a more accurate and better assessment of the associations. Adipocytes are not only fat deposits, but also act as unignorable sites of endocrine secretion, production or regulation, and are substantially involved in the development of low-grade chronic inflammatory status [53,54]. Adiposity is characterized by lipid accumulation, including triacylglycerol and LDL, which will subsequently trigger cellular stress responses and activate inflammation signaling pathways [55]. In addition, abnormal lipid accumulation in obesity is usually accompanied by elevated levels of free fatty acids, this can also trigger a pro-inflammatory cascade, which in turn leads to increased secretion of cytokines, thereby promoting an inflammatory environment [55]. Chronic inflammation status is quite common in the development and persistence of obesity [56,57]. A recent study found that moderate weight gain was also associated with activation of inflammation [57]. Several studies have shown that people with higher BMI have higher CRP levels [58,59], CRP being a typical marker of systemic inflammation, while weight loss reduces CRP levels [60,61]. 

We also found that gender differences in the associations between the inflammation biomarkers and FLD with adiposity, age, smoking, and physical activity were adjusted. Irrespective of body fat percentage, hsCRP level was significantly associated with FLD in female subjects. However, we did not observe significant associations between hsCRP and FLD in males. Gender differences in the association between hsCRP and FLD may be related to sex hormones and different dietary habits between men and women [41]. Previous studies have shown that higher androgen levels in women increase the risk of abdominal obesity and metabolic disorders, while androgen levels in men reduce abdominal obesity, which reduces the risk of FLD [62]. A recent study showed that the protective effect of high blood copper levels on FLD is limited to men and not women and that the body can supplement blood copper by eating meat, seafood, nuts, seeds, and cereals [63]. It is possible that the protective effects of androgens and copper on FLD in men mask the association between hsCRP and FLD, which causes the association between hsCRP and FLD to only be observed in women. For the underlying mechanism of the relationship between hsCRP and FLD, it is still unclear. To date, there has been no evidence that hsCRP can directly lead to hepatocyte steatosis. However, a study conducted in America found that patients subjected to biopsy analysis with confirmed nonalcoholic steatohepatitis had significantly higher plasma hsCRP levels than non-obese healthy subjects and overweight individuals without fatty liver [64]. Additionally, an epidemiology study also showed that NAFLD patients had elevated levels of hsCRP, independent of other metabolic factors [65]. However, a large number of studies have revealed that CRP plays a direct pathogenic role in vascular injury and acts as an important pro-inflammatory factor in the process of atherosclerosis [66]. Therefore, the increase in hsCRP level is likely to promote the occurrence of FLD by promoting the injury of sinusoidal endothelial cells. Another potential mechanism for the CRP response in FLD is the increase in cytokines in the acute phase. For example, IL-6 is an effective stimulator of CRP synthesis. Previous studies reported that compared with healthy individuals, patients with simple steatosis and steatohepatitis had significantly higher serum IL-6 [67].

For the direct association of TNF-α with BF (%) and FLD, no significant associations were observed in the present study, which is inconsistent with previous studies [68,69]. There are several possible reasons: first, there is no association between TNF-α with BF (%) and FLD in the current population; second, the sample size was not large enough to provide enough statistical power for us to detect the potential association. Although this result does not necessarily mean that TNF-α is not a mediator between BF (%) and FLD, future larger sample size studies are needed to clarify their associations.

There are several limitations in the current study. Firstly, physical fitness is an important factor for both obesity and fatty liver diseases [70,71,72], which is not investigated in the present study; however, we added physical activity level [73] as a covariate in the mediation analysis. Secondly, the present study is a cross-sectional study, and as such only association can be concluded, not causal inference. Future cohort or intervention studies should be conducted to verify the findings. Thirdly, osteocalcin produces a widespread increase in insulin sensitivity, and it could be a potential mediator in the production of adiponectin by adipose tissue [74]. This could be a potential research direction for our future studies, but unfortunately, we did not have the data of osteocalcin in the present study. Fourthly, because moderate-intensity/high-intensity physical activity was investigated by questionnaires, this could lead to a recall bias, which needs to be defined more accurately in further studies.

## 5. Conclusions

To conclude, we found that body fat measured by DEXA was significantly associated with a higher risk of FLD in both men and women. Notably, serum hsCRP is a significant mediator in the association between body fat percentage and FLD in females but not in males. These findings provide new research directions for exploring the underlying mechanisms of obesity-related FLD and imply that an anti-inflammatory diet or medicine could be a possible future research direction for overweight or obese women to prevent/control the development or progression of FLD. Additionally, our findings have the potential to improve sex-specific prevention and control strategies for FLD among overweight and obese adults.

## Figures and Tables

**Table 1:** Basic Characteristics of Subjects.

Variable	Total		Male		Female		19–35 Years		36–56 Years	
	n	Median (IQR)/%	n	Median (IQR)/%	n	Median (IQR)/%	n	Median (IQR)/%	n	Median (IQR)/%
**Age (yrs)**	1221	35.0 (30.0, 43.0)	455	33.0 (29.0, 39.0) *	766	37.0 (30.0, 45.0) *	645	30.0 (27.0, 32.0) #	576	44.0 (40.0, 50.0) #
**Height (cm)**	1221	163.7 (158.1, 171.1)	455	173.0 (169.1, 177.6) *	766	159.4 (155.8, 163.5) *	645	166.3 (160.0, 173.2) #	576	161.2 (156.4, 168.0) #
**Weight (kg)**	1221	77.0 (68.9, 88.8)	455	88.4 (78.7, 99.6) *	766	71.9 (65.9, 79.8) *	645	80.8 (72.1, 93.5) #	576	73.5 (66.5, 82.2) #
**BMI (kg/m^2^)**	1221	28.5 (26.3, 31.4)	455	29.4 (26.9, 32.3) *	766	28.0 (26.1, 30.9) *	645	29.2 (26.6, 32.1) #	576	27.9 (26.1, 30.6) #
**Fat mass (kg)**	1221	28.8 (24.8, 34.6)	455	28.8 (24.0, 35.4)	766	28.8 (25.1, 34.2)	645	30.4 (25.7, 36.2) #	576	27.4 (24.2, 32.3) #
**Skeletal muscle mass (kg)**	1221	45.3 (40.2, 54.1)	455	56.5 (52.2, 62.8) *	766	41.4 (38.5, 44.6) *	645	48.0 (41.4, 56.8) #	576	43.3 (39.2, 50.8) #
**BF (%)**	1221	39.3 (34.9, 42.9)	455	33.8 (30.9, 36.8) *	766	41.5 (38.8, 44.5) *	645	39.2 (34.6, 43.3)	576	39.3 (35.2, 42.6)
**hsCRP (mg/L) ^a^**	1215	1.1 (0.5, 2.2)	454	1.0 (0.6, 2.1)	761	1.1 (0.5, 2.3)	641	1.2 (0.5, 2.40)	574	1.0 (0.5, 2.0)
**TNF-α (pg/mL) ^a^**	896	16.5 (10.6, 30.0)	312	16.7 (11.3, 27.8)	584	16.3 (10.2, 31.0)	421	17.3 (11.0, 31.0)	475	16.1 (10.1, 28.9)
**APN (ng/mL)**	896	2426.7 (1595.4, 3371.4)	312	2037.6 (1379.9, 2949.5)	584	2648.8 (1761.9, 3654.3) *	421	2335.6 (1525.0, 3200.6)	475	2495.5 (1676.8, 3526.3)
**Cigarette Smoking in the past week**										
No	694	76.7%	147	46.7% *	547	92.7% *	319	75.2%	375	78.0%
Yes	211	23.3%	168	53.3% *	43	7.3% *	105	24.8%	106	22.0%
**Alcohol Drinking**										
No	695	76.8%	163	51.7% *	532	90.2% *	321	75.7%	374	77.8%
Yes	210	23.2%	152	48.3% *	58	9.8% *	103	24.3%	107	22.2%
**Moderate-intensity physical activity ^b^**										
No	623	68.8%	208	66.0%	415	70.3%	286	67.5%	337	70.1%
Yes	282	31.2%	107	34.0%	175	29.7%	138	32.5%	144	29.9%
**High-intensity physical activity ^b^**										
No	742	82.0%	247	78.4% *	495	83.9% *	342	80.7%	400	83.2%
Yes	163	18.0%	68	21.6% *	95	16.1% *	82	19.3%	81	16.8%
**FLD**										
No	299	24.8%	69	15.4% *	230	30.3% *	178	27.8% #	121	21.4% #
Yes	908	75.2%	378	84.6% *	530	69.7% *	463	72.2% #	445	78.6% #

SD, standard deviation; IQR, interquartile range; BMI, body mass index; BF (%), body fat percentage; hsCRP, high sensitivity C-reactive protein; TNF-α, tumor necrosis factor-α; APN, adiponectin; FLD, Fatty liver disease. ^a^ Mann–Whitney U test. ^b^ moderate- or high-intensity physical activity, Yes, >once per week, No, ≤once per week. * *p* < 0.05 for Student’s *t*-test, Mann–Whitney U test or chi-square test between male and female. # *p* < 0.05 for Student’s *t*-test, Mann–Whitney U test or chi-square test between 19–35 years and 36–56 years.

**Table 2:** Mediation Analysis in the total population and stratified by genders.

Model	Effect	Estimate	SE	Z	*p*
**Total**					
Initial: BF (%)	Total: BF (%) on FLD ^a^	0.2405	0.0250	9.6268	<0.0001
Mediator: hsCRP	BF (%) on hsCRP ^b^	0.1847	0.0245	7.5300	<0.0001
Outcome: FLD	hsCRP on FLD given BF (%) ^c^	0.1812	0.0625	2.9000	0.0037
	Indirect: BF (%) on FLD ^d^	0.0335	0.0125	2.6857	0.0072
	Direct: BF (%) on FLD given hsCRP ^e^	0.2220	0.0257	8.6263	<0.0001
Initial: BF (%)	Total: BF (%) on FLD ^a^	0.2429	0.0251	9.6815	<0.0001
Mediator: TNF-α	BF (%) on TNF-α ^b^	−0.5442	1.1595	−0.4693	0.6390
Outcome: FLD	TNF-α on FLD given BF (%) ^c^	0.0009	0.0008	1.1321	0.2576
	Indirect: BF (%) on FLD ^d^	−0.0005	0.0015	−0.3359	0.7370
	Direct: BF (%) on FLD given TNF-α ^e^	0.2438	0.0252	9.6845	<0.0001
Initial: BF (%)	Total: BF (%) on FLD ^a^	0.2429	0.0251	9.6815	<0.0001
Mediator: APN	BF (%) on APN ^b^	7.3130	11.6965	0.6252	0.5320
Outcome: FLD	APN on FLD given BF (%) ^c^	−0.0003	0.0001	−4.3838	<0.0001
	Indirect: BF (%) on FLD ^d^	−0.0019	0.0031	−0.6038	0.5460
	Direct: BF (%) on FLD given APN ^e^	0.2501	0.0255	9.7900	<0.0001
**Male**					
Initial: BF (%)	Total: BF (%) on FLD ^a^	0.3297	0.0561	5.8802	<0.0001
Mediator: hsCRP	BF (%) on hsCRP ^b^	0.2014	0.0439	4.5844	<0.0001
Outcome: FLD	hsCRP on FLD given BF (%) ^c^	0.4800	0.2556	1.8783	0.0603
	Indirect: BF (%) on FLD ^d^	0.0967	0.0567	1.7037	0.0884
	Direct: BF (%) on FLD given hsCRP ^e^	0.2985	0.0565	5.2836	<0.0001
Initial: BF (%)	Total: BF (%) on FLD ^a^	0.3287	0.0561	5.8589	<0.0001
Mediator: TNF-α	BF (%) on TNF-α ^b^	−1.2564	1.2003	−1.0467	0.2961
Outcome: FLD	TNF-α on FLD given BF (%) ^c^	0.0006	0.0018	0.3546	0.7229
	Indirect: BF (%) on FLD ^d^	−0.0008	0.0032	−0.2490	0.8033
	Direct: BF (%) on FLD given TNF-α ^e^	0.3291	0.0561	5.8677	<0.0001
Initial: BF (%)	Total: BF (%) on FLD ^a^	0.3287	0.0561	5.8589	<0.0001
Mediator: APN	BF (%) on APN ^b^	13.7954	16.3957	0.8414	0.4008
Outcome: FLD	APN on FLD given BF (%) ^c^	−0.0001	0.0001	−0.8838	0.3768
	Indirect: BF (%) on FLD ^d^	−0.0016	0.0034	−0.4714	0.6347
	Direct: BF (%) on FLD given APN ^e^	0.3336	0.0569	5.8662	<0.0001
**Female**					
Initial: BF (%)	Total: BF (%) on FLD ^a^	0.2130	0.0284	7.4941	<0.0001
Mediator: hsCRP	BF (%) on hsCRP ^b^	0.1804	0.0295	6.1109	<0.0001
Outcome: FLD	hsCRP on FLD given BF (%) ^c^	0.1609	0.0632	2.5464	0.0109
	Indirect: BF (%) on FLD ^d^	0.0290	0.0125	2.3241	0.0201
	Direct: BF (%) on FLD given hsCRP ^e^	0.1939	0.0294	6.5876	<0.0001
Initial: BF (%)	Total: BF (%) on FLD ^a^	0.2165	0.0285	7.5927	<0.0001
Mediator: TNF-α	BF (%) on TNF-α ^b^	−0.0605	1.7425	−0.0347	0.9723
Outcome: FLD	TNF-α on FLD given BF (%) ^c^	0.0010	0.0010	1.0473	0.2949
	Indirect: BF (%) on FLD ^d^	−0.0001	0.0025	−0.0251	0.9800
	Direct: BF (%) on FLD given TNF-α ^e^	0.2174	0.0287	7.5870	<0.0001
Initial: BF (%)	Total: BF (%) on FLD ^a^	0.2165	0.0285	7.5927	<0.0001
Mediator: APN	BF (%) on APN ^b^	0.3482	16.215	0.0215	0.9829
Outcome: FLD	APN on FLD given BF (%) ^c^	−0.0003	0.0001	−4.4527	<0.0001
	Indirect: BF (%) on FLD ^d^	−0.0001	0.0050	−0.0210	0.9833
	Direct: BF (%) on FLD given APN ^e^	0.2230	0.0290	7.6782	<0.0001

SE, standard error; BF (%), body fat percentage; FLD, Fatty liver disease; hsCRP, high sensitivity C-reactive protein, TNF-α, tumor necrosis factor-α; APN, adiponectin. All the results were adjusted for age, gender, smoking, drinking and physical activity. ^a^ Represents the total effect of the initial variable on the outcome variable. ^b^ Represents the effect of the initial variable on the mediator variable. ^c^ Represents the effect of the mediator variable on the outcome variable, controlling for the initial variable. ^d^ Represents the indirect effect of the initial variable on the outcome variable through the mediator variable. ^e^ Represents the direct effect of the initial variable on the outcome variable, controlling for the mediator variable.

**Table 3:** Results of Mediation Analysis stratified by age group.

Model	Effect	Estimate	SE	Z	*p*
**19–35 years**					
Initial: BF (%)	Total: BF (%) on FLD ^a^	0.2795	0.0370	7.5600	<0.0001
Mediator: hsCRP	BF (%) on hsCRP ^b^	0.2264	0.0380	5.9559	<0.0001
Outcome: FLD	hsCRP on FLD given BF (%) ^c^	0.0581	0.0606	0.9602	0.3369
	Indirect: BF (%) on FLD ^d^	0.0132	0.0141	0.9352	0.3497
	Direct: BF (%) on FLD given hsCRP ^e^	0.2707	0.0379	7.1500	<0.0001
Initial: BF (%)	Total: BF (%) on FLD ^a^	0.2743	0.0365	7.5214	<0.0001
Mediator: TNF-α	BF (%) on TNF-α ^b^	−0.6699	2.0690	−0.3238	0.7463
Outcome: FLD	TNF-α on FLD given BF (%) ^c^	0.0008	0.0008	1.0200	0.3077
	Indirect: BF (%) on FLD ^d^	−0.0006	0.0024	−0.2255	0.8216
	Direct: BF (%) on FLD given TNF-α ^e^	0.2764	0.0367	7.5226	<0.0001
Initial: BF (%)	Total: BF (%) on FLD ^a^	0.2743	0.0365	7.5214	<0.0001
Mediator: APN	BF (%) on APN ^b^	0.5105	15.1970	0.3626	0.7171
Outcome: FLD	APN on FLD given BF (%) ^c^	−0.0002	0.0001	−1.9225	0.0545
	Indirect: BF (%) on FLD ^d^	−0.0009	0.0029	−0.3173	0.7510
	Direct: BF (%) on FLD given APN ^e^	0.2782	0.0368	7.5512	<0.0001
**36–56 years**					
Initial: BF (%)	Total: BF (%) on FLD ^a^	0.2030	0.0349	5.8166	<0.0001
Mediator: hsCRP	BF (%) on hsCRP ^b^	0.1222	0.0315	3.8813	0.0001
Outcome: FLD	hsCRP on FLD given BF (%) ^c^	0.6370	0.1639	3.8876	0.0001
	Indirect: BF (%) on FLD ^d^	0.0778	0.0288	2.7023	0.0069
	Direct: BF (%) on FLD given hsCRP ^e^	0.1733	0.0363	4.7771	<0.0001
Initial: BF (%)	Total: BF (%) on FLD ^a^	0.2128	0.0355	5.9919	<0.0001
Mediator: TNF-α	BF (%) on TNF-α ^b^	−0.5210	0.9814	−0.5308	0.5958
Outcome: FLD	TNF-α on FLD given BF (%) ^c^	0.0012	0.0023	0.5281	0.5975
	Indirect: BF (%) on FLD ^d^	−0.0006	0.0028	−0.2244	0.8225
	Direct: BF (%) on FLD given TNF-α ^e^	0.2129	0.0355	5.9929	<0.0001
Initial: BF (%)	Total: BF (%) on FLD ^a^	0.2128	0.0355	5.9919	<0.0001
Mediator: APN	BF (%) on APN ^b^	11.9545	18.4330	0.6485	0.5170
Outcome: FLD	APN on FLD given BF (%) ^c^	−0.0003	0.0001	−3.8816	0.0001
	Indirect: BF (%) on FLD ^d^	−0.0037	0.0060	−0.6200	0.5353
	Direct: BF (%) on FLD given APN ^e^	0.2273	0.0369	6.1688	<0.0001

SE, standard error; BF (%), body fat percentage; FLD, Fatty liver disease; hsCRP, high sensitivity C-reactive protein, TNF-α, tumor necrosis factor-α; APN, adiponectin. All the results were adjusted for age, gender, smoking, drinking and physical activity. ^a^ Represents the total effect of the initial variable on the outcome variable. ^b^ Represents the effect of the initial variable on the mediator variable. ^c^ Represents the effect of the mediator variable on the outcome variable, controlling for the initial variable. ^d^ Represents the indirect effect of the initial variable on the outcome variable through the mediator variable. ^e^ Represents the direct effect of the initial variable on the outcome variable, controlling for the mediator variable.

## Data Availability

The data generated for this study are available upon reasonable request to the corresponding author (yangyide@hunnu.edu.cn).
